# Book Review: The Deviant's War: The Homosexual vs. the United States of America

**DOI:** 10.3389/fsoc.2021.667576

**Published:** 2021-03-30

**Authors:** Alex Siu Wing Chan

**Affiliations:** Department of Applied Social Sciences, The Hong Kong Polytechnic University, Hong Kong, Hong Kong

**Keywords:** homosexual rights movement, LGBTQ, civil rights movement, sexual minorities, social inclusion and exclusion

 Eric Cervini (New York, NY: Farrar, Straus and Giroux), 2020, 512 pages, ISBN: 978-0374139797

In 1957, Frank Kameny, a promising scientist employed by the U.S. Defense Department in Hawaii, was instructed to return instantly to Washington, D.C. The Department had grounds to suspect that he was homosexual and eventually fired him. However, different from many others in the same position, Kameny defended his own rights. On the basis of personal observations, previously undisclosed FBI documents, and 40,000 sensitive files, The Deviant's War by Eric Cervini reveals that in the 1960s, the Mattachine Society of Washington was established by Kameny to challenge the regular harassment of homosexual public servants. It explores the overlooked links that connect homosexual rights to the civil rights movement, the political movement, the lesbian movement, and hostility toward transgender people. Most importantly, the tale marked a racial and sexual turning point in American history.

Nowadays, this basic statement is standard advice in developed urban centers around the globe. Yet virtually no knowledgeable individuals accepted it in the 1960s, regardless of their sexual identity. A smart, controversial and incredibly resilient person did something unprecedented to change how the majority of people thought. He was called Frank Kameny, a Harvard-trained mathematician. Kameny might be credited for more important progressive reforms than anyone from the United States after the Second World War, yet that is generally recognized merely by scholars of homosexual culture. Kameny grew up in New York, where he led a decent life. As a talented teenager, he enrolled at Queens College at 16. He realized his interest in his male classmates soon. Similar to almost any homosexual teenager of his age, he believed that these impulses would eventually be forgotten as he developed “usual” love for young women. What made him special was that as he figured that his homosexual urges would never fade, he had to prove to the public that what he did was in fact acceptable.

Cervini reports the prosecution of around 1 million gay or lesbian individuals in the U.S. from 1945 to 1960. This reveals the fact that tremendous time and resources were squandered and numerous lives were lost. By the end of the 1940s, Washington officials announced the “Sex Perversion Elimination Program.” Labeled as “perverts” and “deviants” (which are terms widely used by the media at that time), a vast number of homosexual workers were dismissed following the signing of an executive order in 1953 by President Dwight Eisenhower prohibiting the recruitment of homosexuals in the public sector. In 1957, the American Civil Liberties Union also announced that it was unable to assess the social justification for legislation intended for the oppression or exclusion of sexual minorities.

In 1943, Kameny entered the forces. Following the battle in Europe, he was convinced that he was to be sent to the Pacific, only to find that President Harry Truman delivered two nuclear bombs that called a halt to the battle in Asia too. Kameny believed Truman did the right thing. As a doctor of astronomy, Kameny was employed by the Army Map Service in 1957. The space race soon started following the launch of the first-ever manmade satellite by the Soviet Union. It seemed that Kameny could now fulfill his aspirations of becoming an astronaut. However, his ambitions were soon crushed as the Army learned that he had previously been convicted on a morals charge and dismissed him right away. Kameny opposed his dismissal, different from any other homosexual public servants. He demanded the Supreme Court investigate his case and stated that homosexuals were a community as large as the black population and that the authorities had wrongly labeled him as “dishonest” and “immoral.” It was a groundbreaking declaration.

Kameny was defeated in his lawsuit, but his message had been heard. He persisted with his fight for decades to come by expanding the Mattachine Society, coordinating homosexual strike action, advocating equal employment opportunity for the homosexual community, and changing the mindset of psychiatrists in the United States.

The black civil rights era was the basis and trigger of the transgender movement. According to Cervini, “it was no coincidence that Kameny first made the comparison between homosexual and racial discrimination in the summer of 1960.” That year, adolescent African Americans organized an unprecedented protest in Greensboro. A year later, Kameny informed the chairperson of the federal Civil Service Commission that there was a lot of similarity between the homosexual community and African Americans in the 1920s in the United States. In 1962, Robert Nix, an African American legislator, asked Kameny to come to his office, something which had never occurred before.

One important foundation of this persuasive story was the way Kameny effectively imitated the pioneers of black liberation. He even proposed “Gay Is Good” just like how Stokely Carmichael proposed “Black Is Beautiful.” The work also illustrates the division between people such as Kameny who believed that homosexuals ought to behave like heterosexuals to gain approval (such as wearing appropriate clothing) and others who were more inclined to embrace homosexual identities. Moreover, Cervini accurately recognizes the significance of the Kinsey article, whose findings regarding the existence of homosexual behavior created an outrage in 1948, particularly in the context of the discriminatory therapeutic system.

Most importantly, Kameny transformed humankind by doing two things: telling homosexuals that there was nothing wrong with them and having thousands of heterosexual people to accept that perspective. His absolute best accomplishment was being able to pressure the American Psychiatric Association into recognizing homosexuality as normal behavior in 1973, a remarkable achievement that rendered all additional lesbian, gay, bisexual, and transgender and queer (LGBTQ) development feasible.

The Deviant's War: The Homosexual vs. the United States of America is the story of how a minority community kept fighting for their own rights in the face of tremendous obstacles. Cervini does a really good job of keeping readers entertained throughout the entire book by introducing them to a different side of American history. Thus, the book is highly successful in delivering its objectives. In fact, given that LGBTQ people are becoming progressively active, respected and noticeable members of the community, The Deviant's War is of great significance as it reveals the roots and impact of the LGBTQ activism. I highly recommend this book as it advocates the idea of social equality in modern culture. Apart from the vibrant description of Kameny, the author also gives credit to some other advocates, such as Jack Nichols, Barbara Gittings, and Sylvia Rivera.

## Author Contributions

This whole book review is prepared by AC.

## Conflict of Interest

The author declares that the research was conducted in the absence of any commercial or financial relationships that could be construed as a potential conflict of interest.

